# IUC26687-94 Clinical and Pathologic Outcomes of Resection of Thoracic Residual Masses in Germ Cell Tumors

**DOI:** 10.1093/oncolo/oyag286.031

**Published:** 2026-08-21

**Authors:** S Jubran, E Erbetta, V Verzeletti, A Di Marco, C Pittarello, D Bimbatti, F Pierantoni, M Maruzzo, A Dell'amore, U Basso

**Affiliations:** Department of Surgery, Oncology, & Gastroenterology, University of Padova, Padova, and Oncology Unit 1, Veneto Institute of Oncology IOV-IRCCS, Padova - Italy; Department of Surgery, Oncology, & Gastroenterology, University of Padova, Padova, and Oncology Unit 1, Veneto Institute of Oncology IOV-IRCCS, Padova - Italy; Department of Cardiac, Thoracic, Vascular Sciences and Public Health, University of Padova, Padova - Italy; Department of Surgery, Oncology, & Gastroenterology, University of Padova, Padova, and Oncology Unit 1, Veneto Institute of Oncology IOV-IRCCS, Padova - Italy; Department of Surgery, Oncology, & Gastroenterology, University of Padova, Padova, and Oncology Unit 1, Veneto Institute of Oncology IOV-IRCCS, Padova - Italy; Oncology Unit 1, Veneto Institute of Oncology IOV-IRCCS, Padova - Italy; Oncology Unit 3, Veneto Institute of Oncology IOV-IRCCS, Padova - Italy; Oncology Unit 3, Veneto Institute of Oncology IOV-IRCCS, Padova - Italy; Department of Cardiac, Thoracic, Vascular Sciences and Public Health, University of Padova, Padova - Italy; Oncology Unit 1, Veneto Institute of Oncology IOV-IRCCS, Padova - Italy

## Abstract

**Background:**

Germ cell tumors (GCTs) may involve the lungs and/or mediastinal lymph nodes as metastases from primary testicular or primary mediastinal GCTs (PT-GCTs and PM-GCTs), with different prognoses. After cytoreductive chemotherapy, thoracic residual disease >1 cm is usually resected when feasible. Post-surgical management remains uncertain, especially when a viable tumor is detected.

**Methods:**

We retrospectively reviewed 39 male patients who underwent resection of residual disease in the lungs and/or mediastinum after systemic chemotherapy for GCT at a single tertiary referral center. Twenty-six had metastatic PT-GCTs (66.6%) and 13 PM-GCTs (33.3%). All PT-GCTs had non-seminoma histology, whereas the PM-GCT cohort included one patient with seminoma. Analyses were restricted to 36 months to account for heterogeneous PFS and OS follow-up.

**Results:**

Median age at surgery was 33 years for PT-GCTs and 24 years for PM-GCTs. Median follow-up was 87.6 and 37.2 months, respectively. Surgical approach differed between groups, with thoracoscopic procedures more frequent in PT-GCTs and open approaches in PM-GCTs. Pathologic findings of thoracic specimens also differed: in PT-GCTs, necrosis/fibrosis was reported in 8 patients (30.7%), teratoma in 11 (42.3%), and viable tumour in 7 (27.0%), whereas in PM-GCTs the same findings were reported in 3 (23.1%), 2 (15.4%), and 8 (61.5%), respectively. Within the PT-GCT cohort, teratoma in the primary tumour showed a trend toward association with the presence of teratoma also in metastasectomy samples (p  =  0.097). PM-GCTs demonstrated significantly inferior OS compared with PT-GCTs over the entire follow-up (61.5% vs 86.7% at 7 years, p  =  0.038) and within the first 36 months (61.5 vs 92.1% at 3 years, p  =  0.019). Viable tumour cells were independently associated with inferior OS (HR 11.60, 95% CI 1.15-116.49; p  =  0.037) and PFS (HR 10.06, 95% CI 1.13-89.79; p  =  0.039) in the 36-month multivariable analysis.

**Conclusions:**

Among patients with metastatic GCT undergoing resection of residual thoracic masses, viable tumor at thoracic histology identifies a high-risk subgroup for early progression and shorter survival, particularly within the first three years. The primary mediastinal site also correlates with poorer outcomes. Thoracic resection, therefore, provides clinically relevant prognostic information that may improve post-surgical risk stratification and support tailored postoperative management, including treatment intensification in selected high-risk patients.

